# Supplementary material for: Towards precision ecology: Relationships of multiple sampling methods quantifying abundance for comparisons among studies
Source: PLoS One. 2022 Jun 15;17(6):e0263487. doi: 10.1371/journal.pone.0263487 (PMC9200322; doi:10.1371/journal.pone.0263487)

Supplementary Material 1. Transect-level combined counts of tuna lures and card counts showing transects that were (white circles) and weren’t (black circles) sampled with pitfall traps.


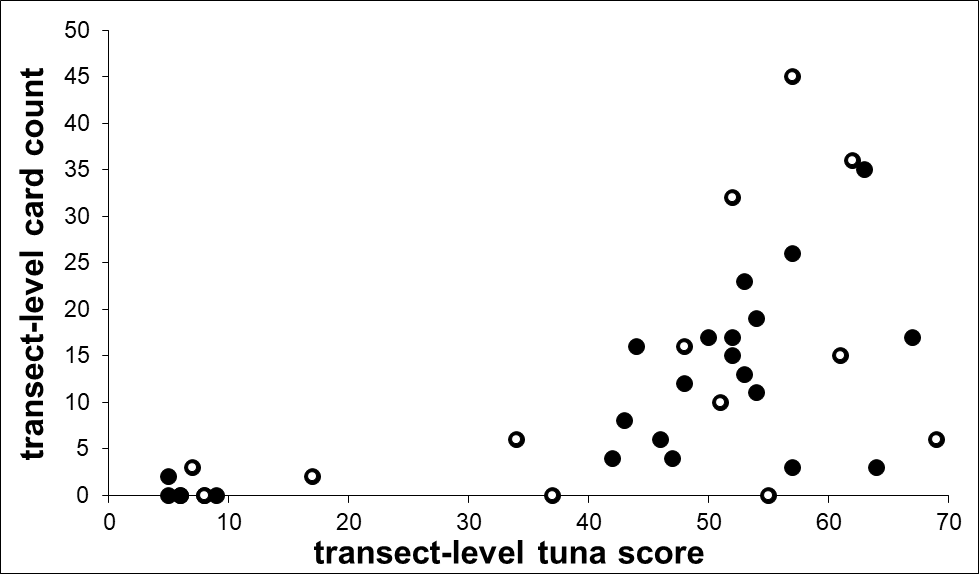

Supplement: S1 File — (DOCX) [file pone.0263487.s001.docx]
